# Increased prevalence of Parkinson's disease in alkaptonuria

**DOI:** 10.1002/jmd2.12367

**Published:** 2023-05-11

**Authors:** Lakshminarayan Ranganath, Milad Khedr, Anna M. Milan, Andrew S. Davison, Brendan P. Norman, Mirian C. H. Janssen, Edward Lock, George Bou‐Gharios, James A. Gallagher

**Affiliations:** ^1^ Department of Clinical Biochemistry and Metabolic Medicine Royal Liverpool University Hospital Liverpool UK; ^2^ Department of Musculoskeletal and Ageing Science, Institute of Life Course and Medical Sciences University of Liverpool Liverpool UK; ^3^ Departments of Internal Medicine & Pediatrics Radboud University Nijmegen Medical Centre Nijmegen Netherlands; ^4^ School of Pharmacy and Biomolecular Sciences Liverpool John Moores University Liverpool UK

**Keywords:** alkaptonuria, homogentisic acid, nitisinone, oxidative stress, Parkinson's disease, tyrosine

## Abstract

Amongst a cohort of 88 alkaptonuria (AKU) patients attending the United Kingdom National Alkaptonuria Centre (NAC), four unrelated patients had co‐existing Parkinson's disease (PD). Two of the NAC patients developed PD before receiving nitisinone (NIT) while the other two developed overt PD during NIT therapy. NIT lowers redox‐active homogentisic acid (HGA) and profoundly increases tyrosine (TYR). A further unpublished case of a Dutch patient with AKU and PD on deep brain stimulation is included in this report. A Pubmed search revealed a further five AKU patients with PD, all without NIT usage. The prevalence of PD in AKU in the NAC appears to be nearly 20‐times higher than in the non‐AKU population (*p* < 0.001) even when adjusted for age. We propose that life‐long exposure to redox‐active HGA may account for the higher prevalence of PD in AKU. Furthermore, the appearance of PD in AKU patients during NIT therapy may be due to unmasking dopamine deficiency in susceptible individuals, as a result of the tyrosinaemia during NIT therapy inhibiting the rate‐limiting brain tyrosine hydroxylase.

**Synopsis**

Parkinson's disease is around 20‐times more prevalent in alkaptonuria (AKU). In AKU, Parkinson's disease coexists before and during nitisinone (NIT) therapy. Parkinson''s disease in untreated AKU may result from life‐long oxidative stress due to exposure to homogentisic acid. NIT‐mediated tyrosinaemia in AKU may unmask Parkinson's disease in susceptible individuals by inhibition of brain tyrosine hydroxylase and thereby further decrease dopamine synthesis. NIT may be withdrawn from those AKU patients where it worsens Parkinson''s disease. DOPA therapy in NIT‐treated AKU patients with PD may need higher doses.

## 1 INTRODUCTION

Alkaptonuria (AKU, OMIM 203500) is a rare inherited disorder present from birth, with a frequency of around 1 in 250 000 people in most parts of the world.^1^ Deficiency of homogentisate 1,2 dioxygenase activity (HGD, EC 1.13.11.5) in the phenylalanine (PHE)/tyrosine (TYR) pathway characterises AKU resulting in accumulation of homogentisic acid (HGA).^2^ The accumulating HGA autooxidises to a melanin‐like pigment via a benzoquinone acetate intermediary in a process known as ochronosis, analogous to the formation of eumelanin and phaeomelanin. The brown–black ochronotic pigment deposits in joint and spine cartilage, tendons, ligaments and heart,^3^ reflected in the clinical features namely aortic stenosis, bone fractures, tendon/ligament/muscle ruptures, kyphosis, scoliosis, spinal surgery, and joint replacements.^3^

Over the last two decades there have been significant advances in the knowledge of AKU as well as the regulatory approval of the first disease‐modifying therapy, in 2020. This followed the successful conclusion to a four‐year randomised controlled study on nitisinone (NIT) in AKU entitled Suitability of Nitisinone in Alkaptonuria 2 or SONIA 2.^4^ In a parallel development in 2012, the United Kingdom National Health Service Highly Specialised Services established the National Alkaptonuria Centre (NAC), where adults with AKU have received NIT 2 mg daily as therapy.^5^ The large cohort of 88 patients which has attended the NAC so far has allowed greater opportunities to increase knowledge about the disease and NIT therapy. Increased prevalence of cataracts and vitiligo are two examples of new knowledge resulting from studies of the NAC cohort.^6^, ^7^ We describe four separate unrelated cases of AKU and Parkinson's disease (PD) from the NAC, one from Netherlands, and review the published cases of this combination in the medical literature.

**FIGURE 1:**
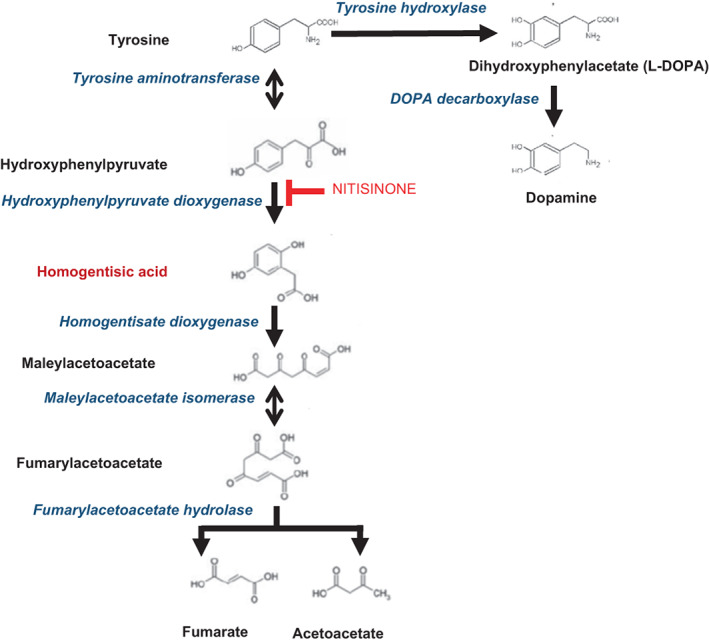
Pathway showing synthesis of DA from TYR as well as the degradation of TYR to fumarate and acetoacetate. Metabolites are shown in black. Enzymes are shown in blue. NIT is shown in red and HGA in brown. DA, dopamine; TYR, tyrosine.

PD is a movement disorder dominated by bradykinesia, rigidity, and tremor due to deficiency of dopamine (DA) in the substantia nigra (SN) neurons. TYR is converted to DOPA by the rate‐limiting enzyme tyrosine hydroxylase (TH), followed by decarboxylation of DOPA to DA (Figure 1). Peripheral DA cannot cross the blood–brain barrier although both TYR and DOPA enter the brain via the large neutral amino acid transporter (LAT‐1).^8^ PD can occur when there is a deficiency of TYR such as in phenylketonuria (PKU).^9^ The TYR/DOPA/DA pathway is tightly regulated to prevent the uncontrolled catecholamines synthesis such as those of DA, adrenaline and noradrenaline. Free cytosolic DA and it's quinone metabolites are oxidants and generate reactive oxygen species (ROS), and are therefore sequestered and stored in vesicles until released in the nigral‐striatal synaptic cleft.^10^, ^11^ In PD increased cytosolic DA due to defective storage in vesicles, as well as the formation of DA quinone (DAQ) from the free cytosolic DA, generate the damaging reactive oxygen species (ROS) and mediate neurotoxicity.^10^, ^11^

**FIGURE 2:**
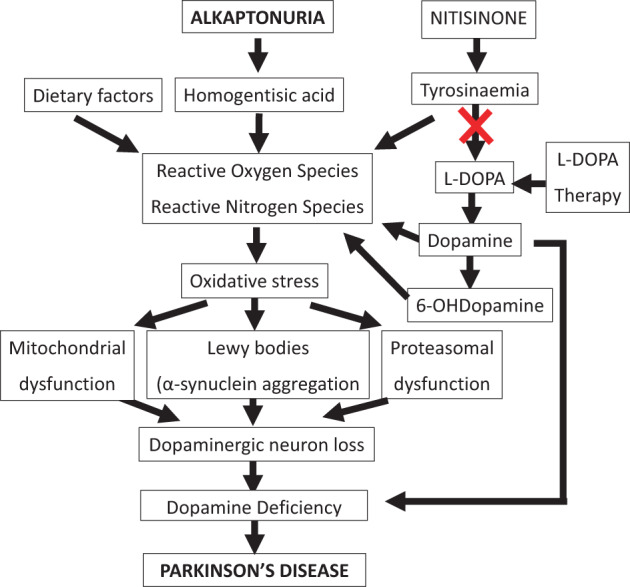
The relationships between NIT‐induced tyrosinaemia and HGA and oxidative stress‐mediated pathophysiology of Parkinson's disease is shown. Autooxidation of homogentisic acid and DA cause oxidative stress which in turn leads to death of substantia nigra neurons, mitochondrial dysfunction and Lewy body formation resulting in Parkinson's disease. Nitisinone‐induced tyrosinaemia leads to oxidative stress as well as inhibits rate‐limiting enzyme tyrosine hydroxylase involved in conversion of TYR to L‐DOPA and subsequently DA. DA, dopamine; DOPA, dihydroxyphenylacetic acid; TYR, tyrosine.

PD is due to progressive dysfunction and death of dopaminergic SN neurons. Oxidative stress drives the pathophysiological process in all forms of PD and is a key factor in the death of SN neurones (Figure 2). During oxidative stress, ROS overwhelm the cellular antioxidant defences leading to dysfunction and damage.^10^, ^11^ In AKU, auto‐oxidative HGA adds to the oxidant stress causing the ‘usual’ sporadic PD in those without AKU; in other words, HGA and non‐HGA oxidant stress may be additive in AKU.^12^ DA deficiency in PD occurs due to lack of synthesis of DA, and inability to store synthesised DA in vesicles in the nigral pre‐synaptic axon terminals, the latter being an earlier event.^13^ Toxicity to SN neurons due to oxidative stress ensuing from free cytosolic DA causes α‐synuclein aggregation, mitochondrial damage, proteosomal dysfunction, Lewy body formation and death of the neuron,^13^ which we propose may be augmented by HGA in the PD of AKU.

## 2 THE NATIONAL ALKAPTONURIA CENTRE COHORT

Eighty‐eight patients with confirmed AKU, through documented elevation in urine homogentisic acid, attended the Royal Liverpool University Hospital (RLUH) between 2012 and 2020. The NAC provided a comprehensive service including NIT 2 mg daily as therapy. No patient received NIT prior to 2012. AKU patients usually visited the NAC once a year.

The data collected from the NAC for Cases 1–4, was approved by the Institutional Audit Committee (Audit No: ACO3836). All procedures followed were in accordance with the ethical standards of the responsible committee on human experimentation (institutional and national) and with the Helsinki Declaration of 1975, as revised in 2000. In addition, the institutional review body (Royal Liverpool University Hospital) explicitly approved the National Alkaptonuria Service audit from which this data was generated. Informed consent was obtained from all patients for inclusion in the study. This is being published as a clinical practice article and standard research ethics process is not therefore appropriate. The data has been anonymised so that the identity of patients is protected.

The data obtained were following standard clinical assessments upon referral to the NAC. Patients were informed verbally and through being supplied with written materials about the activities of the National AKU Service. They were explicitly informed in the patient information booklet of the NAC as follows: ‘We could publish results from the study but if we do, we will make sure you cannot be identified in any way. All data used for publicity or for other research purposes will ensure total anonymity. Please let us know when you are visiting the NAC that you understand this and have no objection to it’.

Netherlands case: an AKU patient from Nijmegen in the Netherlands was diagnosed with PD (Case E, Table 1). Consent was obtained from the patient for the purposes of publication under the assurance that the patient's identity would be protected.

**TABLE 1:** Metabolic Data of AKU patients found to have Parkinson's disease in the NAC (*n* = 88).

	Case 1	Case 2	Case 3	Case 4
sHGA (Pre‐NIT) μmol/L	–	31.3	13.9	41.1
sHGA (NIT) μmol/L	–	3.8	3.2	5.8
uHGA_24_ (Pre‐NIT) μmol/day	20 240	18 619	5175	21 440
uHGA_24_ (NIT)μmol/day	3230	42	128	2212
sTYR (Pre‐NIT) μmol/L	53	48	44	38
sTYR (NIT) μmol/L	880	525	851	841
uTYR_24_ (Pre‐NIT) μmol/day	–	101	5	51
uTYR_24_ (NIT)μmol/day	–	397	309	619
sPHE (Pre‐NIT) μmol/L	–	–	–	65
sPHE (NIT) μmol/L	–	65	44	58
uPHE_24_ (Pre‐NIT) μmol/day		47	–	29
uPHE_24_ (NIT) μmol/day	–	10	11	25
sNIT μmol/L	1.3	1.0	0.6	0.9

Medline search: Pubmed was used to search for terms which included ‘alkaptonuria’ and ‘Parkinson's disease’. Alternative terms such as ochronosis and Parkinsonism were also used in the literature search. Five case reports of PD and AKU were identified in this manner and relevant data for these five cases were extracted from available sources (Cases A–D and F) as shown in Table 1.

## 3 CASE REPORTS

Case 1 A 63‐year‐old White British man with advanced AKU, characterised by extensive AKU morbidity was diagnosed with PD at age 53 years. He started taking over‐the‐counter ascorbic acid 1 g daily since age 58 years. His medications at baseline included warfarin, loperamide, calogen emulsion, ranolazine, digoxin, bisoprolol, lansoprazole, cephalexin, vernagel, paracetamol, senna, docusate, ascorbic acid and stalevo 200 tds (levodopa, carbidopa and entacapone combination). Further details and metabolic data are shown in Tables 1 and 2. He started NIT 2 mg at age 63 years remaining on it until his demise at age 65 years following a sigmoid volvulus.

Case 2 An 82‐year‐old postmenopausal White British woman had advanced AKU developed mild PD characterised by bilateral tremors and rigidity at age 81 years having been on homogentisic acid‐lowering NIT therapy since the age of 72 years. At her recent visit, she was on NIT 2 mg (since July 2012), simvastatin, calcium and vitamin D3, multivitamins, low‐dose aspirin, denosumab, previous zoledronic acid, ascorbic acid, and co‐beneldopa (since May 2021). Her metabolic data are shown in Tables 1 and 2.

Case 3 A 74‐year‐old post‐menopausal White British woman was diagnosed with advanced AKU presented to the NAC for the first time at age 68 years with Parkinsonian features such as bilateral tremor in her hands, bradykinesia and rigidity. Between age 68 and 70 years when she was on NIT 2 mg daily, she became more immobile due to a combination of knee damage and Parkinson's disease, despite the NIT‐induced tyrosinaemia (Tables 1 and 2). She started co‐beneldopa at age 70 years after 2 years of NIT 2 mg therapy due to worsening PD. Her medications at her last visit consisted of NIT 2 mg (since age 68 years), lansoprazole, epilim, gabapentin, nabumetone, amitriptyline, co‐beneldopa, trihexyphenidyl, buprenorphine transdermal patches, calcium and vitamin D3, and PHE/TYR‐free amino acid supplements. Her metabolic data are shown in Tables 1 and 2. She died from myocardial infarction at age 75 years.

Case 4 A British South Asian man of age 67 years was diagnosed with AKU at age 66 years. He displayed bilateral ear and scleral ochronotic pigmentation, as well as advanced AKU. He started taking over‐the‐counter ascorbic acid 1 g daily from age 66 years on his own. Physical examination including his central nervous system at presentation aged 67 years was otherwise normal. His medication at first assessment consisted of citalopram, low‐dose aspirin, ramipril, atorvastatin, bisoprolol, gabapentin, metformin, vitamin D3 and topical piroxicam gel. He remains on NIT 2 mg since first visit at age 67 years. His metabolic and other data are shown in Tables 1 and 2. At 68 years of age he was diagnosed with PD when he developed bilateral tremors in his hands and increased rigidity; he then commenced co‐careldopa 50 mg which he remains on at his most recent review.

**TABLE 2:** Clinical Data of AKU patients found to have Parkinson's disease in the NAC (*n* = 88).

	Case 1	Case 2	Case 3	Case 4
Age at PD diagnosis years	53	80	69	70
Ethnicity	White British	White British	White British	British Indian
Sex	M	F	F	M
Parkinsonian medication	Co‐Careldopa	Co‐Beneldopa	Co‐Beneldopa	Co‐Careldopa
Tremor	Y	Y	Y	Y
Rigidity	Y	Y	Y	Y
Posture abnormality	Y	N	N	N
Gait	Y	N	N	N
Speech	Y	N	N	N
Onset PD pre‐nitisinone	Y	N	Y	N
Onset PD during nitisinone	N	Y – 8 years	N	Y – 3 years
Ascorbic acid	Y	Y	N	Y
Cerebrovascular disease	Y	Y	N	N
Deceased	Y – 65 years	N	Y – age 74	N
Cause of death	Volvulus, further CVA	NA	Myocardial infarction	NA
Miscellaneous	Strokes post PD onset Atrial Fibrillation Embolic strokes	Stroke prior to PD onset Stroke peri‐aortic valve replacement	None	None

Abbreviations: PD, Parkinson's disease; CVA, cerebrovascular accident.

Case E (Nijmegen, Netherlands): A White Dutch postmenopausal woman aged 60 years with advanced AKU characterised by severe arthropathy developed moderate PD characterised by bilateral tremors and rigidity at age 52 years. Her PD was very severe at age 60 years and she was hardly able to walk or speak. Her father developed PD at age 60 years of age even though he did not have AKU. She was not on any medications and refused NIT therapy when offered. Her urine HGA was 4361 μmol/mmol creatinine. Her serum TYR was within the reference range at 93 μmol/L. When she tried levodopa/carbidopa therapy, her PD worsened, and she was commenced on deep brain stimulation (DBS) which is having a positive effect on her PD including her tremor (Table 3).

**TABLE 3:** Data obtained from non‐NAC cases of alkaptonuria and Parkinson's disease showing demographic and clinical data.

	Case A	Case B	Case C	Case D	Case E	Case F
Year published	1968	1969	1970	1995	2016	2020
Reference	14	15	16	19	NA	20
Age years	67	53	52	64	60	42
Age at PD diagnosis years	NK	NK	50	54	52	37
Ethnicity	NK	NK	NK	White French	White Dutch	Turkish
Sex	M	F	F	F	F	F
PD medication	dl DOPA	L‐DOPA	NA	L‐DOPA	DBS*	L‐DOPA
Tremor	NK	NK	Y	Y	Y	Y
Rigidity	NK	Y	Y	Y	Y	NK
Posture	NK	NK	Y	Y	Y	NK
Poor Gait	NK		Y	Y	Y	NK
Speech	NK	NK	NK	N	Y	NK
Onset pre‐nitisinone	Y	Y	Y	Y	Y	Y
Other	Increased HGA, Black urine	Ochronosis, Increased HGA	Family history PD, Severe PD, Ochronosis	Ochronosis	Ochronosis, Family history PD, Severe PD	Depression, Mania, Urinary incontinence

Abbreviations: DBS, deep brain stimulation; HGA, homogentisic acid; NK, not known; PD, Parkinson's disease.

* PD in Case E worsened on L‐DOPA/Carbidopa combination and switched to deep brain stimulation or DBS.

## 4 DISCUSSION

The association between AKU and PD appears to have been first reported in 1968.^14^ Since then single sporadic case reports of this association have appeared in the medical literature.^15^, ^16^, ^17^, ^18^, ^19^, ^20^ The opportunity to address the possible causality of this association of AKU and PD became available to us through access to the relatively large NAC cohort. The prevalence of AKU in non‐consanguineous populations is around 1 in 250 000,^1^ whereas the frequency of PD is much higher at around 1 in 500 in the general population^21^ and 1% in those 65 years of age and older.^22^ In the 88 AKU patients seen in the NAC, there were four with PD, making the prevalence 4.5%, around 20‐times the prevalence in the general population of 0.2%; furthermore, the four AKU patients with PD in the NAC were 65 years of age and older, out of 21 aged 65 years and older, a frequency of 19‐times higher than in the general non‐AKU population. This increased prevalence of PD in AKU suggests that it is probably not coincidental (*p* < 0.001; Chi‐square calculation in Table 4). In addition, men have a higher prevalence of PD compared to women with a male/female ratio of 1.5 in the non‐AKU population,^23^ but this ratio was found to be 0.43 in the 10 known cases of AKU and PD.

**TABLE 4:** Chi‐square tests using data from non‐AKU general population (NHS Inform—Ref. 21) and NAC for numbers of those with and without Parkinson's disease (Graphpad Prism 6.01).

	Parkinson's disease	No Parkinson's disease	Totals
Non‐AKU General population (Ref. 21)	1	499	500
NAC data	4	84	88
Totals	5	583	588

*Note*: The chi‐square statistic is 16.76. The *p*‐value is 0.0001. The chi‐square statistic with Yates correction is 12.0. The *p*‐value is 0.0005.

Two of the four NAC patients developed PD prior to NIT therapy. There were two males and two females with PD in the NAC cohort (Tables 1 and 2). The youngest patient in the NAC to develop PD was a male in his early fifties prior to NIT therapy, while the oldest was a female in her eighties who developed PD after nearly 9 years of NIT therapy. Three of the four NAC patients were also on ascorbic acid raising doubts about the efficacy of the antioxidant to protect against PD despite some in vitro reports that ascorbic acid and N‐acetylcysteine protect against oxidant stress,^24^ although the duration and compliance with ascorbic acid therapy in the NAC patients are unknown. The male who developed PD in his fifties also had the most severe Parkinsonism; he also suffered critical hypotensive episodes during previous joint replacement surgery such that further surgery was deemed too risky. The hypotensive episodes could be due to L‐dihydroxyphenylacetic acid (L‐DOPA) therapy or to the peripheral autonomic nervous system damage from 6‐hydroxydopamine (6‐OHDA), a toxic metabolite generated from L‐DOPA.^25^ The degree of increase in serum or urine HGA could not explain why only these four NAC patients developed PD, although these snapshot measurements cannot fully reflect lifelong exposure to HGA (Tables 1 and 2).

We identified five cases of PD and AKU, four females and 1 male, in the medical literature all pre‐NIT. PD was diagnosed after the age of 60 years in these five cases except for Case F, diagnosed at age 37 years. Case C and E had a family history of PD; a genetic cause for PD is probable in these two cases but unproven. Case E in a female of 56 years from Netherlands is an unpublished case who developed PD at age 52 years with very severe progressive PD, refractory of L‐DOPA therapy. All of these patients, except Case E, received treatment with L‐DOPA; case E worsened on L‐DOPA and received DBS instead and is responding well to it. Nine of the 10 AKU patients described here developed PD well after middle age suggesting that they were due to enhancement of the usual sporadic age‐related PD pathophysiological processes.

In the current analysis, 8 out of the 10 patients were found to develop PD prior to NIT and suggests that HGA, increased from birth, might be the contributory if not causal. It was suggested that HGA was the cause of PD in AKU in the 1960's due to HGA being an electron‐donor producing oxidative stress within the brain.^18^ Circulating HGA has been shown to cause systemic oxidative stress^12^, ^26^; systemic oxidative stress has been implicated in brain damage including PD.^27^ In the SONIA 1 and SONIA 2 clinical studies, increased inflammation and oxidative stress was described.^28^ Fatal haemolytic anaemia due to oxidative damage and methaemoglobinaemia in untreated AKU has been reported.^29^ Therefore, there is evidence from studies that oxidative stress is factual in AKU. Additionally, lifelong attempts to consume lower amounts of protein in AKU patients has been associated with malnutrition and the effect of this malnutrition on antioxidant defences is unknown and can add to the oxidant stress in AKU.^30^ HGA is converted to melanin‐like pigment in a process termed ochronosis,^3^ as a result of which nigro‐striatal ochronosis was proposed to cause PD in AKU^17^, ^18^; however, no ochronosis has been observed during post‐mortem examination of the brain of patients with AKU, even though ochronosis of the dura was observed.^31^, ^32^ No HGA has been detected in the CSF or the brain of AKU mice,^33^, ^34^ suggesting that HGA in AKU contributes to PD through effects outside the brain. This could imply that ROS produced outside the brain could affect the brain by being transported through the bollod‐brain barrier.

Two of the four NAC patients, cases 2 and 4, developed PD during NIT therapy on the background of lifelong exposure to HGA. Further, case 3 worsened despite NIT‐induced tyrosinaemia necessitating L‐DOPA therapy; these three cases suggest that NIT therapy did not decrease risk of PD by reducing HGA, while not excluding the possibility that NIT‐induced tyrosinaemia may have unmasked PD by worsening the DA deficiency. Inhibition of the enzyme 4‐hydroxyphenylpyruvate dioxygenase by NIT blocks the PHE/TYR pathway and causes marked tyrosinaemia (Table 1; Figure 1). Tyrosine is known to produce oxidant stress itself and, in the NIT‐treated patients, tyrosinaemia could cause additional oxidant stress.^35^ Additionally, it has been suggested that NIT‐induced tyrosinaemia could increase tyrosine flux down alternate pathways including the DA pathway and formed the rationale to develop NIT as an anti‐Parkinsonian therapy^36^; beneficial effects of spill over from NIT‐induced tyrosinaemia have been described in oculocutaneous albinism B and advanced neuroblastoma.^37^, ^38^

**FIGURE 3:**
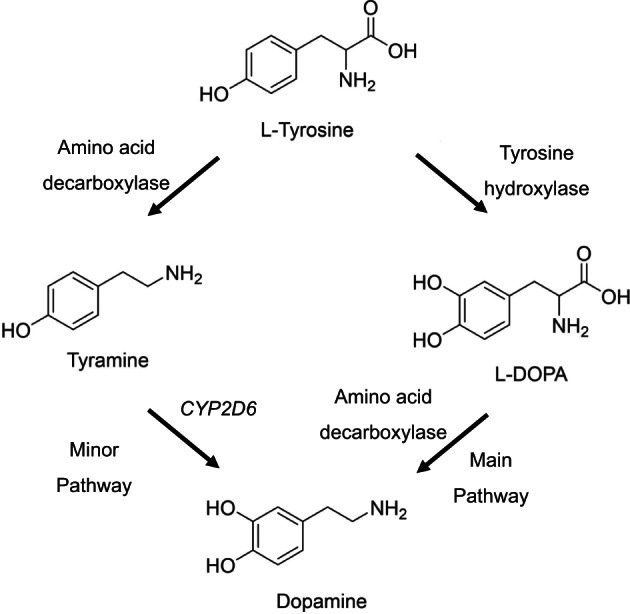
The pathways of formation of DA from TYR are shown including the enzymes catalysing the reactions. The main pathway is through conversion of TYR to L‐DOPA and then DA effected by rate‐limiting tyrosine hydroxylase and amino acid decarboxylase sequentially. The alternative pathway involves conversion to tyramine and then DA effected by amino acid decarboxylase and oxidoreductase *CYP2DS* sequentially. The minor pathway may adapt during decreased tyrosine hydroxylase activity. DA, dopamine; DOPA, dihydroxyphenylacetic acid; TYR, tyrosine.

However, recent studies of rodent NIT‐induced tyrosinaemia on brain tyrosine metabolism questions whether NIT can be beneficial in PD of AKU. NIT administered to AKU mice produced tyrosinaemia similar to humans.^33^, ^34^, ^39^ Examination of the CSF of NIT‐treated AKU mice found the expected markedly increased TYR concentrations.^33^ Two further studies in NIT‐treated AKU mice found that the brain TYR and tyramine were increased by around 10‐ and 25‐times compared with the untreated; despite the increase in brain TYR, *DA in the brain did not increase and instead tended to decrease*, suggesting that the rate‐limiting enzyme TH responsible for the conversion of TYR to L‐DOPA was likely inhibited by the tyrosinaemia to prevent excessive catecholamine formation, as shown previously.^34^, ^39^, ^40^ Tyramine is synthesised from brain TYR in situ since tyramine like dopamine cannot cross the blood–brain barrier. Since tyramine was much increased in the brains of these mice, and since tyramine can be converted to DA via an alternate pathway, this mechanism could explain why more severe DA deficit was prevented (Figure 3).^41^ It is also therefore not surprising that the development of NIT as an anti‐Parkinsonian has failed since DA decreased in the brain,^36^ and also since TH deficiency is a known cause of PD.^42^ We therefore suggest that tyrosinaemia during NIT administration could produce DA deficiency by inhibition of TH and unmask subclinical dopamine deficiency. If PD worsens significantly after NIT in AKU, and cannot be overcome by higher doses of L‐DOPA therapy, there is a case for withdrawing NIT; however, NIT is beneficial for most other features of AKU and a decision to withdraw NIT must be made on a case by case basis.

In addition to the auto‐oxidative nature of HGA, TYR, DA and DA quinone, especially in a prooxidant environment, DA can be converted to the more neurotoxic 6‐OHDA, known to cause neuronal death and lead to PD and is a well‐established animal model of PD.^43^ Direct measurement of 6‐OHDA was not carried out in the mouse studies. The 6‐OHDA generation may be important both before and during L‐DOPA therapy of PD and explain the transient benefit of L‐DOPA as PD therapy since L‐DOPA therapy may not only be replenishing the brain with DA but causing death to the SN neurons.^44^

**FIGURE 4:**
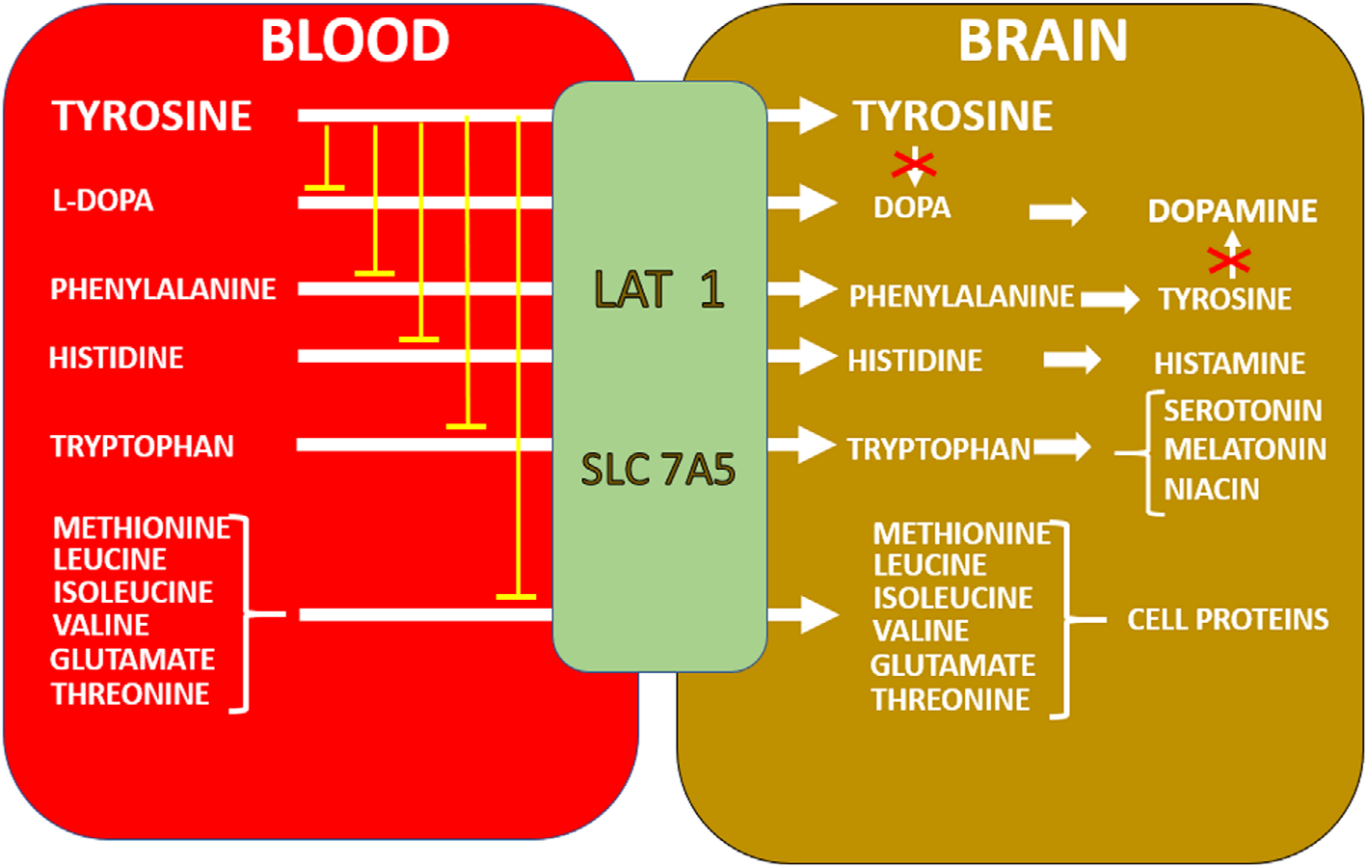
NIT‐induced tyrosinaemia and effect on LAT 1 (SLC 7A5). Markedly increased circulating TYR competes with L‐DOPA and other essential and non‐essential amino acids for transport from the blood into the brain, decreasing their transport into the brain (shown as yellow lines from TYR label). During tyrosinaemia consequent to increase in brain TYR, tyrosine hydroxylase is inhibited (shown as red cross) preventing conversion of brain TYR (directly transported as well as derived from PHE) to L‐DOPA and therefore to DA. Tyrosinaemia therefore decreases formation of DA in the brain from TYR, as well as decreases transport of L‐DOPA into the brain, attesting to multiple defects in Parkinson's disease of AKU during nitisinone therapy. DA, dopamine; DOPA, dihydroxyphenylacetic acid; TYR, tyrosine.

All NAC patients were on L‐DOPA combination therapy with peripheral DOPA decarboxylase inhibitors to decrease peripheral metabolism of L‐DOPA and increase brain availability of L‐DOPA. Both TYR and L‐DOPA are transported into the brain across the blood–brain barrier by LAT‐1.^8^ Nothing is known about the competition between L‐DOPA and TYR for the LAT‐1 *in tyrosinaemia*, genetic or acquired, even though PD in PKU has been attributed to competition between PHE and TYR for LAT‐1.^9^ The advice for PD patients on L‐DOPA therapy is to eat smaller protein meals to minimise competition between L‐DOPA and other neutral L‐amino acids such as TYR (Figure 4). Competition between L‐DOPA and TYR during NIT‐induced tyrosinaemia, where circulating TYR more than 15‐times higher during NIT compared to Pre‐NIT, could potentially decrease the availability of L‐DOPA in the SN, and require higher L‐DOPA doses. The issue of DA formation during L‐DOPA therapy for PD while also receiving NIT for AKU should be clarified by further studies.

There are limits to what we know about PD in AKU. It is not known if there is a more general sub‐threshold DA deficit in AKU, as has been described in PKU.^9^ It is not known whether HGA or TYR is more important as an oxidant stressor since it would shed light on whether earlier HGA‐lowering by NIT would be better for AKU patients despite tyrosinaemia from the PD perspective. The dose–response of tyrosinaemia to oxidant stress needs to be characterised. More attention to helping patients comply with supervised lower‐protein diet during NIT would be worthwhile, as ideally, lowering of HGA with minimal increase in TYR while also addressing the issue of maintaining anti‐oxidant nutritional defences should be done in AKU patients without PD with a view to prevention. While the co‐existence of PD and AKU both prior to and during NIT therapy suggests that pre‐existing exposure to HGA could be important in causing PD, it is not known if NIT‐induced tyrosinaemia alone could cause PD, an important issue for children and young people with Hereditary Tyrosinaemia type 1 (HT‐1) currently treated with NIT. Under‐reporting of cases of PD in AKU is likely and better data collection is needed to resolve these issues. Consideration must be given to provide protective antioxidant therapies such as ascorbic acid and N‐acetylcysteine for AKU and possibly HT‐1 patients from an early age, whose diets are probably compromised from long‐term attempts at dietary modifications.

In conclusion, we believe that this review of the literature and our own experience as part of the NAC illustrates that the co‐existence of PD and AKU is not coincidental given the very high frequency of PD in AKU as well as sound pathophysiological basis discussed here. Lifelong HGA exposure in untreated AKU likely accelerates the natural history of PD. NIT‐induced tyrosinaemia in AKU may worsen the DA deficit by inhibiting TH and unmask subthreshold PD in AKU necessitating L‐DOPA therapy. NIT‐treated AKU patients with PD may need higher L‐DOPA doses due to competition between L‐DOPA and TYR for LAT‐1.

## AUTHOR CONTRIBUTIONS

Lakshminarayan Ranganath assessed the patients, recognised connection between AKU and Parkinson's disease and wrote the manuscript. Milad Khedr assessed the patients, and edited the manuscript. Anna M. Milan, Andrew S. Davison and Brendan P. Norman carried out the chemical analyses and edited the manuscript. Mirian CH Janssen provided a case and edited the manuscript. Edward Lock, George Bou‐Gharios and James A. Gallagher provided scientific input and edited the manuscript.

## FUNDING INFORMATION

This work was supported by funding granted in April 2012 by the NHS England Highly Specialised Services in establishing the UK National Alkaptonuria Centre in the Royal Liverpool University Hospital. The funding source was not involved in the study design, collection, analysis and interpretation of data, the writing of the manuscript, or in the decision to submit the manuscript for publication. The authors confirm independence from the funders; the content of the article has not been influenced by the funders.

## CONFLICT OF INTEREST STATEMENT

Lakshminarayan Ranganath, Milad Khedr, Anna M. Milan, Andrew S. Davison, Brendan P. Norman, Mirian CH Janssen, Edward Lock, George Bou‐Gharios, James A. Gallagher have no conflict of interest to declare.

## ETHICS STATEMENT

All procedures followed were in accordance with the ethical standards of the responsible committee on human experimentation (institutional and national) and with the Helsinki Declaration of 1975, as revised in 2000. The data collected from the NAC for cases 1 to 4, was approved by the Institutional Audit Committee (Audit No: ACO3836). Informed consent for Case E was obtained in Nijmegen, Netherlands.

## Data Availability

The authors agree to honour any reasonable request by other researchers for materials, methods or data necessary to verify the conclusion of the article.
